# What a Sham(e): Sham‐Controlled Conditioned Pain Modulation Effects on Pressure but Not Heat Pain Thresholds in Healthy Volunteers

**DOI:** 10.1002/ejp.70067

**Published:** 2025-07-01

**Authors:** Madeleine Hau, Laura Sirucek, Iara De Schoenmacker, Robin Lütolf, Lindsay Gorrell, Michèle Hubli, Petra Schweinhardt

**Affiliations:** ^1^ Department of Chiropractic Medicine, Integrative Spinal Research Group, Balgrist University Hospital University of Zurich Zurich Switzerland; ^2^ Neuroscience Center Zurich University of Zurich Zurich Switzerland; ^3^ Center for Neuroplasticity and Pain (CNAP), Department of Health Science and Technology Aalborg University Aalborg Denmark; ^4^ Spinal Cord Injury Center, Balgrist University Hospital University of Zurich Zurich Switzerland; ^5^ Biomedical Data Science Lab, Institute of Translational Medicine Swiss Federal Institute of Technology (ETH) Zurich Zurich Switzerland

## Abstract

**Background:**

Conditioned pain modulation (CPM) is a well‐established experimental paradigm to study descending pain modulation in humans, measuring the pain modulatory effect of a painful conditioning stimulus (CS) on a painful test stimulus (TS). Control conditions using a non‐painful CS accounting for modulatory effects not attributable to the painfulness of the CS are seldom included. Thus, this study aimed to differentiate CPM effects from perceived changes of the TS unrelated to the painfulness of the CS by comparing effects of a painful and a control CS on four different TS.

**Methods:**

Forty‐nine healthy participants underwent a combined parallel and sequential CPM paradigm with a cold water bath (median NRS 8/10) as painful and an ambient‐temperature sham water bath (median NRS 0/10) as non‐painful control CS. TS were pressure and heat pain thresholds (PPT, HPT) (parallel and sequential) and temporal summation of pain (TSP, sequential) (pressure and heat). Larger TS changes with the painful compared to the control CS were interpreted as sham‐controlled CPM effects.

**Results:**

A parallel sham‐controlled CPM effect was only detected for PPT (significantly larger PPT increases during the painful compared to the control condition (*p* = 0.009)). HPT increased for both conditions without a significant difference between conditions (*p* = 0.152). TSP was successfully induced but not modulated by either CS (*p* > 0.05).

**Conclusion:**

This study demonstrates sham‐controlled CPM effects on PPT, but not on HPT, most likely due to heat adaptation or habituation. This challenges the interpretation of prior studies using CPM paradigms with HPT as TS without a control condition.

**Significance:**

This study highlights the importance of including control conditions in CPM paradigms using HPT as TS. HPT increased similarly during a painful and a control condition, most likely due to adaptation and habituation. Although these are known effects, CPM studies rarely control for them. Pressure pain thresholds increased more during the painful than during the control condition, making it a more suitable TS, especially when a control condition is absent.

## 1 Introduction

Nociceptive processing is influenced by descending pain modulation, shaping the subjective experience of pain. Animal studies discovered that a strong noxious stimulus can trigger the descending pain inhibitory system leading to an analgesic effect. This “pain inhibits pain” phenomenon was termed diffuse noxious inhibitory controls (DNIC) (Le Bars et al. 1979).

Conditioned pain modulation (CPM) refers to paradigms that are routinely used to study DNIC‐like effects in humans. Typical CPM paradigms consist of a tonic painful conditioning stimulus (CS) to trigger descending pain inhibition, and a second painful test stimulus (TS) applied to a remote body site (Yarnitsky et al. 2015). The change in the TS perception induced by the CS represents the CPM effect (Pud et al. 2009).

A large variety of CPM paradigms exists utilising CS and TS of different modalities (Kennedy et al. 2016; Nuwailati et al. 2022). Common CS are cold/hot water baths and tonic painful pressure, while supra‐threshold pain stimulation and pressure or heat pain thresholds (PPT, HPT) are frequently used TS (Kennedy et al. 2016; Nuwailati et al. 2022). CPM effects on temporal summation of pain (TSP), supposedly interacting on a spinal level (Le Bars et al. 1979; Price and Dubner 1977), have also been investigated (Horn‐Hofmann et al. 2018). The magnitude and test–retest reliability of CPM effects differ depending on the CS–TS combination and the painfulness of the CS, with growing consensus that cold water baths or ischemic pain as CS and pressure stimuli as TS show high intra‐session reliability (Imai et al. 2016; Nuwailati et al. 2022; Razavi et al. 2014). Nevertheless, a methodological limitation of the existing CPM literature is that only a few studies employ control conditions using a non‐painful CS (Cummins et al. 2021; Kennedy et al. 2020; Lütolf et al. 2022). Studies lacking a control condition do not control for potential changes in the perception of the TS that cannot be attributed to the painfulness of the CS, including adaptation, habituation, sensitisation or distraction (which may still be stronger during a painful than non‐painful CS). This might increase the risk of false‐positive findings because changes in the perception of the TS during or after the CS might be interpreted as CPM effects despite being related to mechanisms other than descending inhibition induced by a painful stimulus. Thus, including a control CS is necessary for robust conclusions regarding the nature of the observed pain modulatory mechanism. However, the existing expert consensus on CPM lacks a recommendation for control conditions while suggesting using different TS modalities (Yarnitsky et al. 2015). To further probe the importance of using a control condition with different TS modalities, the present study compared pain modulatory effects of a cold water bath as a painful CS and an ambient‐temperature sham water bath as a non‐painful control CS for four different TS (PPT, HPT, and pressure and heat TSP) in healthy subjects.

## 2 Methods

### 2.1 General

The present study was part of a larger project within the framework of the Clinical Research Priority Program (CRPP) Pain at the University of Zurich (https://www.crpp‐pain.uzh.ch/en.html) investigating pain phenotypes of patients with chronic pain. In this study, data of the healthy control cohort are presented. The CRPP project consisted of three visits, each lasting approximately 3 h. The CPM paradigm was performed during the second visit. Prior to the CPM paradigm, a neurophysiological assessment including pain‐evoked potentials was performed. Because results of the pain‐evoked potentials are not relevant for the research question of this study, they will not be further discussed. The study was approved by the local ethics committee and was performed according to the guidelines of the Helsinki Declaration (EK‐04/2006/PB_2016‐02051 and PB_2019‐00136, clinicaltrial.gov number: NCT02138344 and NCT04433299).

### 2.2 Participants

Participants aged between 18 and 80 years were consecutively recruited from November 2019 until April 2022 via online advertisements and oral communication. The sample size was targeted to answer the research question of the larger project. Exclusion criteria were any chronic pain condition, low back pain on more than 3 consecutive days in the last year, any major medical or psychiatric condition that affects physical capacity or pain sensitivity (e.g., severe heart disease, diabetes, autoimmune disorder, major depressive disorder, chronic pain condition), pregnancy or inability to follow study instructions. The participants were individually age‐ and sex‐matched to a cohort of chronic pain patients. All participants gave their written informed consent before participation. Demographic information (age, sex, height and weight) and participant characteristics that might affect participant performance or that have been associated with variations in individual CPM responses (body mass index (BMI), smoking status, and physical activity) were collected (Naugle et al. 2017; Parkerson et al. 2013). Physical activity was assessed using the International Physical Activity Questionnaire (IPAQ), calculating a physical activity score based on the self‐reported duration and frequency of low, moderate, and vigorous physical activity per week (Craig et al. 2003).

### 2.3 Study Overview

Participants underwent a CPM paradigm with a cold water bath as painful CS and a sham water bath as control CS. Both water baths were used in a single session with a break of 10 min between the two conditions. The order of the two water baths was randomised across participants.

The pain modulatory effects of the painful and the control CS were tested on four different TS, namely PPT and HPT, as well as pressure and heat TSP. The CPM paradigm followed a combined parallel, i.e., TS during CS, and sequential, i.e., TS after the end of CS, design. For PPT and HPT, parallel and sequential pain modulatory effects were assessed; for TSP, only sequential pain modulatory effects were assessed due to time constraints during the water bath. Unrelated to the present research question, TSP using pin pricks was assessed once at the beginning of the session prior to the CPM testing.

**FIGURE 1:**
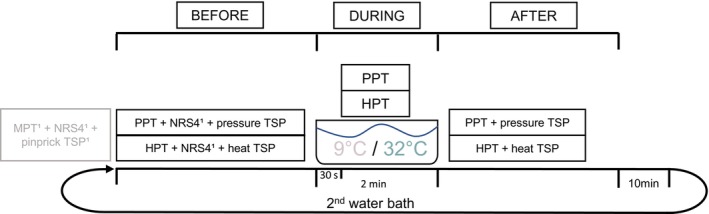
Schematic overview of the study procedure. Grey colour indicates assessments which are not part of the present study. NRS4 refers to the individualised stimulus intensity necessary to induce a perceived pain intensity of 4 out of 10 on a 11‐point numerical rating scale, determined prior to the TSP assessment and applied to evoke TSP. HPT, heat pain threshold; MPT, mechanical pain threshold; PPT, pressure pain threshold; TSP, temporal summation of pain. ^1^Assessment was conducted before the first water bath only.

Participants were familiarised with PPT, HPT and pin pricks during their first visit. Prior to the CPM session, they were additionally familiarised with the TSP procedure using pin prick stimuli and standardised instructions. Seven different examiners were involved in the data collection. All examiners were trained in all procedures used during this study and had either obtained a quantitative sensory testing (QST) certificate issued by the German Research Network on Neuropathic Pain or were trained by study personnel with this certification. An overview of the study procedure is provided in Figure 1.

### 2.4 Numerical Rating Scale

The perceived pain intensity induced by the water baths and the supra‐threshold stimuli during TSP was assessed using an 11‐point numerical rating scale (NRS). The scale was labelled from 0 (“not painful”), 1 (“first sensation of pain”) to 10 (“most intense pain tolerable”). Participants verbally reported their perceived pain intensity and were allowed to use decimal places in their ratings.

### 2.5 Conditioning Stimuli

The painful CS consisted of an iced‐cooled cold water bath with a target temperature of 9°C ± 0.5°C. A second sham water bath with a target temperature of 32°C ± 0.5°C served as the control CS. The temperature was monitored with a thermometer, which was not visible to the participant. The painful and control CS were both applied to the hand contralateral to the TS testing site. Participants were asked to immerse their hand to the wrist into the circulating water for 2 min. The water baths were maintained for the full duration, even if the TS assessment was completed earlier. Participants were allowed to remove their hand if the water bath became unbearably painful but were encouraged to re‐immerse the hand when possible. Pain induced by the water bath was assessed using the NRS at three timepoints: right after immersion, 30 s after immersion and immediately before hand removal. The maximum of the three pain ratings was used for further analyses. Participants were asked about any residual pain from the first water bath before the start of the second condition.

### 2.6 Test Stimuli

PPT and HPT were always assessed before the water bath, 30 s after hand immersion, and following the water bath. The TSP assessment before and after each water bath always followed the corresponding threshold assessment (e.g., pressure TSP immediately after PPT). Given this assessment order, pain thresholds were on average assessed 24.4 s after the end of the water bath for the first and 102.4 s for the second modality, with each assessment lasting on average 25.3 s. TSP assessment started between 48.3 s after the end of the water bath for the first and 129.1 s for the second modality, lasting on average 47.5 s. TS assessments were completed on average within 171 s (maximum: 251 s) after the end of the water bath. The order of pressure and heat assessments was randomised across participants but kept constant across measurement timepoints, i.e., before, during, after the water bath, within each participant.

The TS were applied on the participant's hand. The choice of right or left hand was matched to the hand tested in the patient to whom the participant was individually matched. The hand tested in the patients was either contralateral to the patient's most painful body site or, in the case of low back pain patients, the non‐dominant hand. For the majority of the participants (81.6%), the TS were applied on the left hand.

In addition to being a reliable TS for various CPM paradigms (Imai et al. 2016), PPT was chosen as a TS to examine descending pain modulatory effects on deep afferents. HPT was chosen because it is recommended to include a mechanical and a thermal TS according to current CPM guidelines (Yarnitsky et al. 2015), and to examine descending pain modulatory effects on superficial afferents. TSP was chosen as a temporal dynamic stimulus that is suggested to lead to spinal facilitation at the dorsal horn (Price and Dubner 1977), supposedly interacting with the effects of CPM (Horn‐Hofmann et al. 2018; Sirucek et al. 2020).

#### 2.6.1 Pressure Pain Threshold

Pressure was applied on the participant's thenar eminence, targeting the abductor pollicis brevis muscle, using a hand‐held algometer with a rubber tip of 1 cm in diameter (Pressure Algometer FDN 200 or FDN 100, Wagner Instruments, USA). The PPT was determined using a continuous stimulus increasing at a rate of 0.5 kg/s. Following the standardised instructions of the QST protocol according to the German Research Network on Neuropathic Pain (Rolke et al. 2006), participants were instructed to report as soon as they perceived an additional sensation to pressure. For safety reasons, a maximum pressure of 10 kg/cm^2^ was applied. If a participant's PPT was not reached with the application of 10 kg/cm^2^, they were assigned a value of 10 kg/cm^2^ as their PPT. None of the participants reached the safety cut‐off for the PPT assessment.

#### 2.6.2 Heat Pain Threshold

Heat was applied using a 3 cm × 3 cm Advanced Thermal Stimulation (ATS) thermode (PATHWAY Pain & Sensory Evaluation System, Medoc, Ramat Yishai, Israel) on the web between the thumb and the index finger at the dorsum of the participant's hand. HPT was determined using a continuous stimulus starting at a baseline temperature of 32°C and increasing at a rate of 1°C/s. Following the standardised instructions of the QST protocol according to the German Research Network on Neuropathic Pain (Rolke et al. 2006), participants were instructed to press a button as soon as they perceived an additional sensation to heat. For safety reasons, an automatic cut‐off temperature was set to 50°C. If a participant's HPT was not reached with the application of 50°C, they were assigned a value of 50°C as their HPT. Three participants reached the safety cut‐off for the HPT assessment, two of them only during/after but not before the CS.

#### 2.6.3 Temporal Summation of Pain

TSP was assessed for the two stimulation modalities, i.e., mechanical and thermal. Pressure was applied using the same algometer as for PPT (Cathcart et al. 2009), and heat taps were applied using a Thermal Stimulation Analyser (TSA) II 3 cm × 3 cm ATS thermode (Medoc Ltd., Ramat Yishai, Israel). TSP was induced using a supra‐threshold stimulus intensity that was individually adjusted to a perceived pain intensity of 4 on the NRS (‘NRS4’). The pressure or temperature necessary to reach NRS4 was determined using a staircase method, according to the following protocol: Starting from PPT + 1 kg/cm^2^ or HPT + 2°C, the intensity of pressure and heat stimuli of 1 s duration were increased if the given rating was less than 3.5 or decreased if the rating was more than 4.5 on the NRS until the target rating was reached. If the first rating was within ± 0.5 of the target rating, the same stimulus intensity was repeated, to ensure consistency of the rating. The stimulus intensities for NRS4 were determined immediately before the TSP assessment, before the first water bath, and kept constant throughout the rest of the session.

To assess TSP, participants received 12 stimuli with a duration of 1 s and an inter‐stimulus interval of 2 s on the thenar eminence for pressure TSP and on the web between the thumb and the index finger at the dorsum of the hand for heat TSP. They were asked to report the perceived pain intensity on the NRS after each stimulus. The timing of the stimulus and the inter‐stimulus interval were controlled manually using a metronome. Pressure intensity was controlled by continuously monitoring the scale on the algometer, while heat intensity was set via the target temperature of the thermode. The TSP magnitude was calculated as the pain rating of the last stimulus minus the pain rating of the first stimulus.

### 2.7 Statistical Analysis

Statistical analysis was performed in R using version 4.2.1 for Windows (R Core Team 2022). A *p*‐value < 0.05 was considered statistically significant. Parallel pain modulatory effects induced by the painful or the control CS were defined as the change in the perception of the TS during compared to before CS; sequential ones as the change in the perception of the TS after compared to before CS. Pain modulatory effects are reported as absolute and percentage change, with positive values indicating pain inhibition and negative values indicating pain facilitation.

Normal distribution of raw data or of the residuals of the models, depending on the statistical test used, was evaluated by assessing z‐scores of skew and kurtosis, which were required to be smaller than 2, and by visual assessment of histograms and QQ‐Plots. Data that fulfilled criteria for parametric tests, i.e., PPT and HPT, were analysed using linear mixed‐effects models (lmer() function of the lme4 package). Data obtained using the ordinal NRS, therefore not fulfilling criteria for parametric tests, i.e., TSP, were analysed using cumulative link mixed models (clmm() function of the ordinal package) with a logit link function (Christensen 2018; Liddell and Kruschke 2018). All models were corrected for age and sex by including age and sex as well as their interaction terms with condition and timepoint as the independent variables of interest. To prevent overfitting of the models, age and sex were removed if they had no significant main or interaction effect. In addition, all non‐significant interaction terms were removed from the models to obtain an estimation of the main effects. Missing data points were coded as NA because the linear mixed‐effects models can handle missing data without imputing values.

Pain ratings of the two water baths were compared using a Wilcoxon signed‐rank test for the maximal pain rating for each water bath.

To estimate the modulatory effects on PPT and HPT, the main effects of condition (two levels: painful CS and control CS) and timepoint (three levels: before, during and after water bath), the interaction terms of condition and timepoint, and random effects of participant ID were assessed on the dependent variables PPT and HPT. In the presence of a significant main or interaction effect, post hoc tests were conducted using the emmeans() function and were corrected for multiple comparisons using the Tukey–Kramer correction. To ensure that the models' results were not driven by outliers, all models were assessed for the presence of influential cases, defined as outliers with a large influence on the model (Field et al. 2014). This assessment included the identification of individual data points (not to be confused with participants) that (1) were detected as outliers within the model, i.e., the *z*‐score of the residual was larger than 2 and (2) exerted a large influence on the model, i.e., Cook's distance exceeded a cut‐off value of $\frac{4}{\left(n-k-1\right)}$, with *n* as the number of data points in the model, and *k* as the number of predictors. Finally, it was examined whether the identified data points were driving the statistical inference of the model by re‐running the model without these data points. If the exclusion of the data points changed the statistical inference of the model, i.e., the model's result was driven by a small number of outliers, the results of the model without the data points are reported. Otherwise, the results of the full model are reported. In case data points were excluded, the original model including all data points is reported in Table S1.

For PPT, a significant interaction of condition and timepoint was detected which did not allow for the assessment of the main effects of condition and timepoint because main effects are uninterpretable in the presence of an interaction effect (Field et al. 2014). However, the main effect of timepoint for the painful CS, i.e., the cold water bath, was of relevance for the present study to answer the question whether a pain modulatory effect was present in this condition. Therefore, an additional model following the same procedure as described above was performed with PPT data of the cold water bath only. By chance, the randomisation produced a slight imbalance regarding the order of conditions, i.e., cold water bath first vs. sham water bath first. The imbalanced order of conditions was tested for significance using a binomial test. To analyse whether the order of conditions had an influence on PPT or HPT at ‘baseline’, i.e., before the cold and the sham water bath, PPT and HPT were compared before the first and the second water bath using paired *t*‐tests.

To test whether TSP was induced at ‘baseline’, i.e., before the cold and the sham water bath, the main effect of stimulus number (two levels: first and last) on the dependent variable pain rating was analysed. The model was corrected for differences in TSP magnitude at ‘baseline’, by including condition and the interaction of stimulus number with condition as independent variables. The pain modulatory effect on TSP was estimated by a model evaluating the main effect of condition (two levels: painful CS and control CS) and timepoint (two levels: before and after), and the interaction term of condition and timepoint on the dependent variable TSP magnitude.

For all models assessing pain modulatory effects, a significant condition and timepoint interaction with larger changes in the perception of the TS in response to the painful compared to control CS was interpreted as a sham‐controlled CPM effect.

A power analysis for each model was conducted using a data‐driven, simulation‐based approach (Kumle et al. 2021) (mixedpower package) with 1000 simulations for the actual sample size of 49 participants. To assess if the power was driven by sample size or effect size, additional simulations were performed for hypothetical sample sizes of 100 and 500 participants. A power of 1−*β* ≥ 0.8 was considered sufficient to detect a true positive effect. Results of the power analysis are in Table S2.

To explore interindividual differences in CPM responsiveness, a qualitative and a correlation analysis of individual parallel pain modulatory effects was performed. Details of the analysis and results are provided in Methods S1, Results S1, Figure S1, Figure S2 and Table S3.

## 3 Results

### 3.1 Participants

Of the 62 (35 female and 27 male) recruited participants, one participant was excluded due to the development of a pain condition between inclusion and the first visit. Twelve participants were tested at a different anatomical location than the hand because they were matched to chronic pain patients in whom both hands were affected. Due to sensory differences between body sites and to reduce heterogeneity in the dataset, data from these 12 participants were not included in the present study. Thus, the final cohort consisted of 49 participants. Demographics and participant characteristics shown to be associated with variations in individual CPM response or participant performance (Naugle et al. 2017; Parkerson et al. 2013) are reported in Table 1. There was no systematic difference in these characteristics between participants receiving the painful CS first and participants receiving the non‐painful CS first (BMI: *t*(30.25) = 1.24, *p* = 0.224; IPAQ scores: *W* = 336.5, *p* = 0.295; Smokers: *χ* ^2^ = 2.24, *p* = 0.326), indicating that any potential effect of these characteristics would affect both conditions to a similar degree.

**TABLE 1:** Demographics and participant characteristics.

Number of participants	49
Sex (f:m)	23:26
Age (years)	50.0, SD = 16.04
Weight (kg)	72.1, SD = 12.70
Height (cm)	172.8, SD = 8.74
BMI (kg/m^2^)	24.0, SD = 2.82
Number of smokers	6
Physical activity (IPAQ score)	2392.0 (2967.00)

*Note:* Values are presented as mean and standard deviation (SD) for parametric data and median and interquartile range for non‐parametric data,i.e., IPAQ score.

Abbreviations: BMI, body mass index; f, female; IPAQ, International Physical Activity Questionnaire (Craig et al. 2003); m, male.

### 3.2 Water Baths

Pain ratings during the cold water bath were significantly higher compared to the sham water bath (Table 2), without an order effect on perceived intensity (*p* = 0.450). All participants rated the cold water bath as painful. Five participants reported that they perceived the sham water bath as painful, i.e., reported pain ratings of NRS ≥ 1 (median (interquartile range) of the respective five participants: 2.0 (3.00), maximum: 4.0). As per the influential case analysis described in the Methods section, only one data point from one of these five participants was identified as an influential case affecting the inference of the HPT model. Specifically, the HPT of one participant before the cold water bath was excluded from the HPT model. Three participants removed their hand from the cold water bath prematurely due to unbearable pain (duration of the cold water baths: 60 s, 74 s and 77 s). For the participant with the shortest hand immersion, the TS could not be assessed during the water bath, leading to two missing data points (PPT and HPT during water bath). The reduced duration of the cold water bath did not affect the sequential pain modulatory effects in the respective participants indicated by none of the respective data points being identified as influential cases in any model.

**TABLE 2:** Thresholds and TSP in the painful CS and control CS condition.

		Cold			Sham			Test statistics		
	Pain ratings of water bath median (interquartile range) [NRS]	0 s	30 s	120 s	0 s	30 s	120 s			
		3.0 (3.00)	6.0 (3.00)	8.0 (2.75)	0.0 (0.00)	0.0 (0.00)	0.0 (0.00)	*V* = 1225, *p* < 0.001		
		Before	During	After	Before	During	After	Conditon	Timepoint	Interaction
Model PPT	PPT mean, SD (kg/cm^2^)	4.3, SD = 1.68^a^	5.1, SD = 1.94^a^	4.5, SD = 1.68	4.3, SD = 1.57	4.5, SD = 1.60	4.4, SD = 1.56	Not meaningful^b^		
	Pain modulatory effect (before—during/after)		***0.9 (25%), SD = 1.14 (30.16%)***	0.2 (10.5%), SD = 1.03 (30.02%)		0.2 (8.0%), SD = 0.79 (20.67%)	0.1 (7.0%), SD = 0.90 (24.78%)			*F[238.03] = 4.75 p = 0.009*
Model HPT	HPT mean, SD (°C)	***43.7, SD = 3.60*** ^a^	***46.1, SD = 3.69*** ^a^	***45.6, SD = 3.56***	***43.2, SD = 3.51***	***45.2, SD = 3.44*** ^a^	***45.1, SD = 3.51*** ^a^	*F[219.1] = 17.47 p < 0.001*	*F[219.2] = 92.99 p < 0.001*	
	Pain modulatory effect (before–during/after)		2.1 (4.9%), SD = 1.89 (4.57%)	2.0 (4.6%), SD = 1.59 (3.88%)		2.0 (4.7%), SD = 1.60 (3.88%)	2.0 (4.7%), SD = 1.59 (3.92%)			F[222.3] = 1.90 p = 0.152
Model TSP pressure	Pressure TSP median (interquartile range) [ΔNRS]	2.0 (2.00)		2.0 (3.00)	1.0 (2.00)		1.0 (2.00)	z = 0.83 p = 0.406	z = 0.72 p = 0.473	
	Pain modulatory effect (before–after)			0.0 (2.00) −17.3%, SD = 90.09%			0.0 (2.00) 14.9%, SD = 79.20%			z = −0.33 p = 0.739
Model TSP heat	Heat TSP median (interquartile range) [ΔNRS]	2.0 (3.00)		2.0 (3.00)	2.0 (4.00)		2.0 (2.00)	z = 0.82 p = 0.410	z = 0.99 p = 0.324	
	Pain modulatory effect (before–after)			0.0 (2.00) −0.3%, SD = 81.37%			0.0 (2.00) ‐19.9%, SD = 81.38%			z = 0.07 p = 0.946

*Note:* Italic font indicates significant main/interactions effects of the statistical models; bold and italic font indicates significant post hoc tests. For parametric data (PPT, HPT and % changes) values are presented as mean across subjects, SD and, F and *p* values from linear mixed‐effects models. For non‐parametric data (TSP, pain ratings of both water baths) values are presented as median, interquartile range, and *z* and *p* values from cumulative link mixed models. In all models, raw PPT, HPT, pressure and heat TSP were used as dependent variable. Presented pain modulatory effects represent the interaction effect of timepoint and condition, i.e., differences in pain modulatory effects between the cold and the sham condition. The cold water bath served as painful and the sham water bath as control CS.

Abbreviations: CS, conditioning stimulus; HPT, heat pain threshold; NRS, numerical rating scale; PPT, pressure pain threshold, SD, standard deviation; TSP, temporal summation of pain.

^a^ One missing value.

^b^ Main effects are not meaningful due to the significant interaction effect of condition and timepoint.

There was no significant imbalance in the order of the conditions (cold water bath first vs. sham water bath first: 19 vs. 30, *p* = 0.076). None of the participants reported residual pain from the first water bath before the start of the second water bath.

### 3.3 Significant Sham‐Controlled Parallel CPM Effect on PPT

**FIGURE 2:**
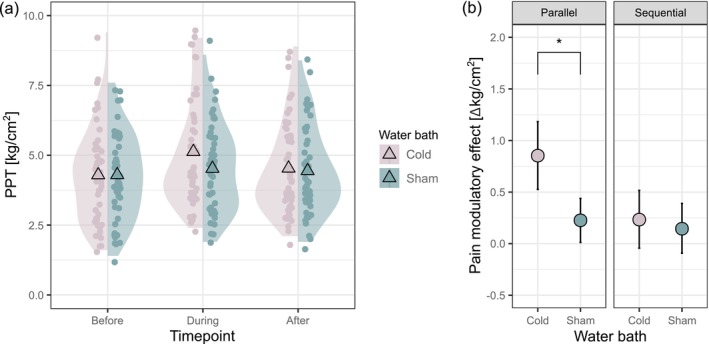
Significant sham‐controlled parallel CPM effect on PPT. (a) PPT before, during and after the cold (painful CS) and the sham (non‐painful control CS) water bath (representing main effects of condition and timepoint), mean PPT (triangle), raw values (dots) and density distribution (cloud) at each timepoint for both water baths. (b) Parallel and sequential pain modulatory effect on PPT (representing interaction effect of condition and timepoint), mean of absolute PPT changes (dots) with 95% CI (error bars) for both water baths. CPM, conditioned pain modulation; PPT, pressure pain threshold; *p < 0.05.

The interaction between condition and timepoint was significant for the PPT model, indicating that the change in PPT, i.e., the pain modulatory effect, differed between the painful and the control CS (Table 2). Post hoc tests showed that there was a higher parallel pain modulatory effect of the painful CS compared to the control CS, indicated by a significantly higher increase of PPT during the cold compared to the sham water bath (*t*(238) = −2.87, *p* = 0.009, Figure 2b). There was no difference in sequential pain modulatory effects between the two CS (*t*(238) = −0.45, *p* = 0.879, Figure 2b). Additionally, there was no significant effect of age or sex (age: *F*[45.98] = 0.10, *p* = 0.753; sex: *F*[45.99] = 2.45, *p* = 0.124). Twenty data points were identified as influential cases, but removal did not change the statistical inference of the model. Therefore, no data points were excluded from the model.

The results of the power analysis showed sufficient power for the detection of parallel sham‐controlled CPM effects with the present sample size of 49 (1−*β* = 0.813). In contrast, for sequential sham‐controlled CPM effects on PPT, the power analysis demonstrated that even for samples of 100 and 500 participants, respectively, only a power of 1−*β* = 0.1 and 1−*β* = 0.289 would be achieved. This indicates that sequential CPM effects were too small to be detectable with a meaningful sample size (Table S2).

There was no significant difference in ‘baseline’ PPT, i.e., PPT before the first and the second water bath, indicating that there was no order effect on PPT (*t*(47) = 0.23, *p* = 0.820).

An additional analysis on the PPT changes during and after the cold water bath only revealed a significant main effect of timepoint (*F*[94.13] = 16.71, *p* < 0.001), indicating a pain modulatory effect of the painful CS on PPT. Post hoc tests showed that there was a parallel but no sequential pain modulatory effect indicated by significantly higher PPT during but not after the cold water bath compared to before (parallel: *b* = −0.85, *t*(94.2) = −5.60, *p* < 0.001; sequential: *b* = −0.24, *t*(94.1) = −1.57, *p* = 0.263).

### 3.4 No Sham‐Controlled CPM Effect on HPT

**FIGURE 3:**
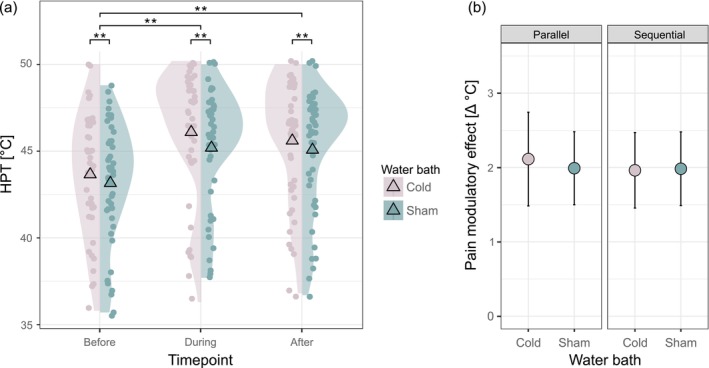
No significant sham‐controlled CPM effect on HPT. (a) HPT before, during and after the cold (painful CS) and the sham (non‐painful control CS) water bath (representing main effects of condition and timepoint), mean HPT (triangle), raw values (dots) and density distribution (cloud) at each timepoint for both water baths. (b) Parallel and sequential pain modulatory effect on HPT (representing interaction effect of condition and timepoint), mean of absolute HPT changes (dots) with 95% CI (error bars) for both water baths. CPM, conditioned pain modulation; HPT, heat pain threshold; **p < 0.001.

There was no significant interaction between condition and timepoint for HPT (Table 2, Figure 3b), indicating that there was no difference in pain modulatory effects on HPT between the painful CS and the control CS. The main effect of timepoint on HPT was significant, indicating that HPT changed over time within each condition (Table 2, Figure 3a). Post hoc tests showed that there were parallel and sequential pain modulatory effects of both CS on HPT, i.e., significant HPT increases during and after both water baths compared to before (parallel: *b* = −2.14, *t*(219) = −12.30, *p* < 0.001; sequential: *b* = −1.97, *t*(219) = −11.44, *p* < 0.001, Figure 3a). In addition, a significant main effect of condition on HPT was observed (Table 2). Post hoc tests showed that HPT was significantly higher throughout the cold compared to the sham water bath (*b* = 0.59, *t*(219) = 4.18, *p* < 0.001). There was no significant effect of age or sex (age: *F*[45.95] = 0.03, *p* = 0.861; sex: *F*[45.99] = 2.23, *p* = 0.143). Removal of 19 data points (6.6%) identified as influential cases changed the statistical inference of the model, i.e., main effect of condition, and thus, the model without the respective data points is reported. The model including all data points can be found in Table S1.

The power analysis showed that even a sample size of 500 participants would not provide sufficient power to detect a significant interaction between condition and timepoint for HPT, indicating that effect size, rather than sample size, drives the power of the model (Table S2).

Analyses on influences of the order of the conditions on ‘baseline’ HPT revealed that HPT before the second water bath was significantly higher compared to the first water bath (*t*(47) = −4.27, *p* < 0.001).

### 3.5 TSP Was Successfully Induced but Not Altered by CPM

The stimulus intensity necessary to induce an NRS4 was 6.3 kg/cm^2^, SD = 2.40 kg/cm^2^ for the pressure TSP and 48.3°C, SD = 2.88°C for the heat TSP.

**FIGURE 4:**
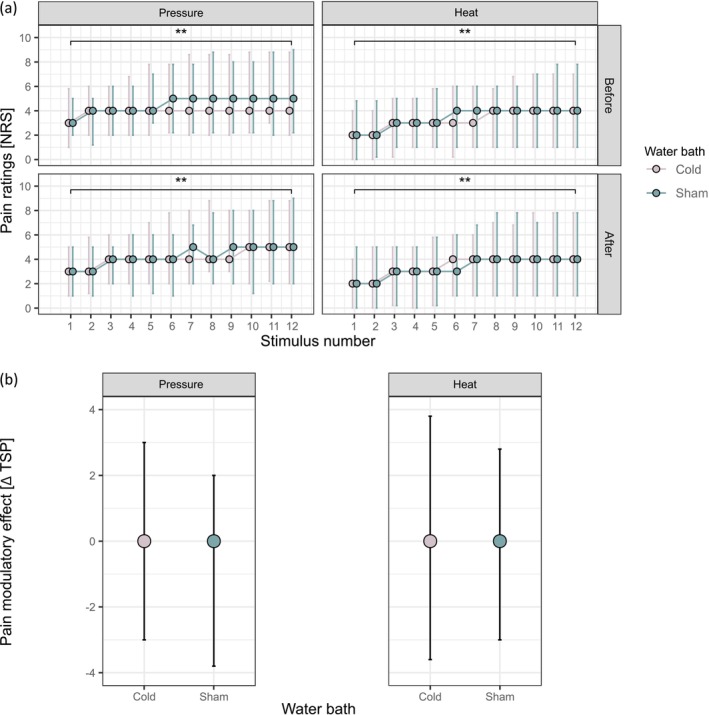
No pain modulatory effect on successfully induced TSP. (a) Time course of pain ratings during the TSP induction before and after each water bath for pressure and heat, median pain ratings on the NRS (dots) with upper and lower quartiles (error bars) for each stimulus number. (b) Sequential pain modulatory effect on pressure and heat TSP, median of absolute TSP changes (dots) with upper and lower quartiles (error bars) for each water bath. TSP, temporal summation of pain; **p < 0.001.

The TSP magnitude did not differ before the cold or sham water bath indicated by the non‐significant interaction between stimulus number and condition (pressure: *b* = 0.13, *z* = 0.49, *p* = 0.621; heat: *b* = 0.10, *z* = 0.40, *p* = 0.692). A significant TSP was induced by pressure and heat stimuli before both water baths indicated by a significant main effect of stimulus number on pain rating (pressure: *b* = 2.65, *z* = 8.04, *p* < 0.001; heat: *b* = 2.77, *z* = 8.34, *p* < 0.001; Figure 4a).

There was no significant (sequential) sham‐controlled CPM effect on pressure nor heat TSP, indicated by the non‐significant interaction terms of condition and timepoint (Table 2, Figure 4b). Further, for both models, there were no significant main effects of condition or timepoint on the TSP magnitude (Table 2, Figure 4b), meaning that neither the painful nor the control CS exerted a pain modulatory effect on TSP. There was no significant effect of age or sex (age: *b* = 0.01, *z* = 0.60, *p* = 0.549; sex: *b* = −1.10, *z* = −1.38, *p* = 0.168 for pressure; age: *b* = −0.01, *z* = −0.23, *p* = 0.820; sex: *b* = 0.54, *z* = 0.53, *p* = 0.593 for heat).

The power analysis showed that even a sample size of 500 participants would not provide sufficient power to detect a significant interaction between condition and timepoint for TSP, indicating that the power of the model was driven by effect size rather than sample size (Table S2).

## 4 Discussion

This study aimed to differentiate CPM effects from changes in the perception of the TS unrelated to the painfulness of the CS by investigating the pain modulatory effects of a cold water bath as painful CS and an ambient‐temperature sham water bath as non‐painful control CS on PPT, HPT, as well as pressure and heat TSP. Only PPT showed larger pain modulatory effects during the painful compared to the non‐painful control CS representing a sham‐controlled CPM effect. HPT increased during and after the cold water bath, but did so similarly during and after the sham water bath. TSP was successfully induced using pressure and heat stimuli, but was not modulated by either CS.

PPT being the only TS which demonstrated a sham‐controlled CPM effect extends previous studies indicating that PPT is a reliable TS with larger pain modulatory effects and lower variability compared to other TS (Imai et al. 2016; Kovacevic et al. 2021; Nuwailati et al. 2022). Contrasting previous findings (Gajsar et al. 2018; Locke et al. 2014; Oono et al. 2011; Peterson et al. 2019), reporting sequential CPM effects 5–10 min after the CS, no sequential pain modulatory effect was detected in the present study, assessed less than 3 min after CS, possibly explained by several factors. Firstly, sequential paradigms produce smaller pain modulatory effects than parallel paradigms (Pud et al. 2009; Sirucek et al. 2020), potentially due to distraction effects present during, but not after, the CS contributing to parallel CPM effects (Defrin et al. 2010; Yarnitsky et al. 2015). Secondly, the CS in the present study was applied on the same spinal segment as the TS. Previous work suggests that segmentally applied CS yield smaller CPM effects compared to extra‐segmentally applied CS (Oono et al. 2011, 2013). As this has not been formally tested, we compiled data from studies using CS or TS on multiple body sites (Table S4), supporting the notion that extra‐segmental CS induce larger pain modulatory effects than segmental CS. Thirdly, participants in the present study were older than in many other CPM studies on healthy volunteers (Nuwailati et al. 2022) and pain modulatory effects decrease with age (Edwards et al. 2003; Pud et al. 2009). These three factors considered, the sequential pain modulatory effects on PPT might have been too small to be detected, further supported by the power analysis.

Contrasting PPT, no sham‐controlled CPM effect was observed for HPT. Only two previous CPM studies using HPT as TS included a control condition. One study did not report any HPT changes in response to a painful nor a control CS (Oono et al. 2013), possibly due to the use of a time‐consuming QST battery as TS and 30‐min‐CS, i.e., a methodologically very different CPM paradigm compared to the present study. The other study reported significant HPT increases during a painful CS and with measuring the HPT repeatedly without any CS to control for repeated‐measures, i.e., perceived TS changes unrelated to the presence of a CS (Cummins et al. 2021). Congruent with the present results, the painful CS induced no CPM effect beyond the repeated‐measures effects.

The lack of a sham‐controlled CPM effect was related to HPT showing significant parallel and sequential pain modulatory effects in response to the cold *and* the sham water bath. Most other studies using HPT as TS reported significant parallel and/or sequential pain modulatory effects (Cummins et al. 2021; Imai et al. 2016; Joshi et al. 2020; Knezevic et al. 2019; Kovacevic et al. 2021; Liebano et al. 2013; Nilsen et al. 2012; Razavi et al. 2014; Schneider et al. 2022; Trouvin et al. 2022; Tuveson et al. 2007; Williams et al. 2013), while only some did not (Kosek and Hansson 1997; Nahman‐Averbuch et al. 2013; Oono et al. 2013). Given that HPT increased similarly in response to the cold and the sham water bath, it is likely that adaptation and habituation (Greene and Hardy 1962; Greffrath et al. 2007; Prescott 1998) contributed to the observed changes of the HPT, rather than true CPM effects. Adaptation of heat sensitive receptors is a peripheral mechanism leading to fast declines in perceived pain intensity, whereas habituation is considered a central process associated with slower and less pronounced declines (Greffrath et al. 2007). Particularly, responses to temperatures around the individual's HPT tend to adapt and habituate (Greffrath et al. 2007; Hashmi and Davis 2010). However, a previous study also showed reduced pain perception of supra‐threshold heat in response to a painful, a control, and no CS, indicating that adaptation and habituation may generalise to other types of thermal TS (Treister et al. 2010). Heat adaptation and habituation might also explain why HPT, but not PPT, showed sequential pain modulatory effects. The presence of habituation is further supported by lower HPT before the first compared to the second water bath. These results indicate that HPT might not only adapt within a single CPM paradigm, but also habituate over the course of longer experimental sessions. Notably, all previous studies using time intervals of less than 1.5 min between the TS assessment before and during/after the CS reported pain modulatory effects on HPT, while pain modulatory effects were less frequent with longer time intervals (Imai et al. 2016; Joshi et al. 2020; Knezevic et al. 2019; Kovacevic et al. 2021; Nahman‐Averbuch et al. 2013; Oono et al. 2013). TS assessments separated by short time intervals are more prone to adaptation processes because C‐fibre responses remain attenuated up to 4 min after heat stimulation at 49°C (LaMotte and Campbell 1978). Moving the thermode between HPT assessments to reduce peripheral adaptation (Liebano et al. 2013; Williams et al. 2013) does not fully replace a control condition because habituation can still lead to a reduction of perceived heat pain intensity by approximately 40% (Greffrath et al. 2007).

Taken together, the ample evidence for adaptation and habituation of repeated heat stimulus responses means that it is impossible to postulate *true* CPM effects on HPT without a control condition. Respective studies increase the risk of false‐positive findings in the literature, as adaptation and habituation effects might be falsely interpreted and reported as CPM. Additionally, even if a control condition is used, the presence of adaptation and habituation might hamper the detection of true CPM effects on HPT due to ceiling effects. Less pronounced adaptation and habituation (Ohrbach and Gale 1989) might explain why a true, sham‐controlled CPM effect was observed for PPT but not HPT. In summary, the present study underlines the importance of control conditions in CPM paradigms and favors PPT over HPT as TS, particularly when a control condition is absent. This raises the question of whether an update of current expert recommendations on CPM testing is warranted, as these do not endorse the use of control conditions and advise the use of both mechanical and thermal TS (Yarnitsky et al. 2015).

In the present study, TSP was successfully induced using pressure and heat stimuli, but not altered by either CS. Animal literature suggests that CPM and TSP act on wide‐dynamic‐range neurons in the spinal cord (Le Bars et al. 1979; Le Bars 2002; Price and Dubner 1977) and are thus supposed to interact. Human studies assessing parallel and sequential CPM effects on TSP are inconsistent (Horn‐Hofmann et al. 2018; Oono et al. 2013; Sirucek et al. 2020; Tousignant‐Laflamme and Marchand 2009), suggesting relatively weak effects, susceptible to random noise, which might explain the present results. Additionally, the same methodological considerations discussed as potential reasons for the absent sequential pain modulatory effects on PPT also apply to TSP. This result might therefore be a matter of methodology rather than of biology.

## 5 Limitations

This study was not primarily designed to evaluate adaptation and habituation processes. Prior to the CPM paradigm, participants underwent an assessment of pain‐evoked potentials. Furthermore, TSP was performed between the HPT/PPT assessments before and during the water baths. Both aspects might have influenced the measured thresholds. However, such influences would be expected in both CPM conditions. Thus, the comparison of sham‐controlled CPM effects on PPT and HPT remains valid, indicating that adaptation and habituation need to be considered in CPM paradigms using HPT. PPT, assessed with the same protocol, was less susceptible to these effects. Additionally, CPM effects on TSP, only assessed sequentially, may have been detectable with a parallel paradigm. Manually controlled pressure intensities during TSP may have varied slightly across stimuli.

## 6 Conclusions

This study highlights the importance of control conditions in CPM paradigms, particularly when using HPT as TS, which increased to a similar degree irrespective of the painfulness of the CS. This indicates the influence of adaptation and habituation processes on HPT, which cannot be disentangled from true CPM effects without a control condition. Misinterpretation of results leads to an increased risk of false‐positive findings and an inability to draw sound conclusions about mechanisms that are supposedly investigated. In contrast, sham‐controlled CPM effects were observed for PPT, emphasising the advantages of PPT as TS, which seems to be less influenced by adaptation and habituation processes.

## Author Contributions

This study was designed by P.S., M.Hu, L.S., I.D., and R.L. The experiments were performed by L.S., I.D., and L.G. The data were analysed by M.Ha and L.S., and the results were critically examined by all authors. M.Ha had a primary role in preparing the manuscript, which was edited by L.S., P.S., M.Hu, I.D, L.G., and R.L. All authors have approved the final version of the manuscript and agree to be accountable for all aspects of the work.

## Disclosure

Use of Artificial Intelligence: A large language model (ChatGPT 4) was used for proofreading and rephrasing.

## Supporting information


Data S1.
