# Supplementary material for: What a Sham(e): Sham‐Controlled Conditioned Pain Modulation Effects on Pressure but Not Heat Pain Thresholds in Healthy Volunteers
Source: Eur J Pain. 2025 Jul 1;29(6):e70067. doi: 10.1002/ejp.70067 (PMC12212405; doi:10.1002/ejp.70067)
Supplement: Supplementary file 1 — Data S1. [file EJP-29-0-s001.docx]

# Supplementary Material

**What a sham(e): Sham-controlled conditioned pain modulation effects on pressure but not heat pain thresholds in healthy volunteers**

Madeleine Hau^1,2^, Laura Sirucek ^1,2,3^, Iara De Schoenmacker^2,4,5^, Robin Lütolf^4^, Lindsay Gorrell^1^, Michèle Hubli^2,4^, Petra Schweinhardt ^1,2^

^1^Department of Chiropractic Medicine, Integrative Spinal Research Group, Balgrist University Hospital, University of Zurich, Zurich, Switzerland

^2^Neuroscience Center Zurich, University of Zurich, Zurich, Switzerland

^3^Center for Neuroplasticity and Pain (CNAP), Department of Health Science and Technology, Aalborg University, Aalborg, Denmark

^4^Spinal Cord Injury Center, Balgrist University Hospital, University of Zurich, Zurich, Switzerland

^5^Biomedical Data Science Lab, Institute of Translational Medicine, Swiss Federal Institute of Technology (ETH) Zurich, 8008 Zurich, Switzerland

Corresponding Author: Madeleine Hau, +41 44 386 57 06, madeleine.hau@balgrist.ch

# Methods S1

# Exploratory analysis on interindividual differences in pain modulatory effects

Individual pain modulatory effects induced by the painful and the control CS, as well as individual sham-controlled CPM effects (pain modulatory effect induced by the painful CS minus pain modulatory effect induced by the control CS), were calculated for each participant. Of note, these individual pain modulatory effects refer to any change in the TS and not to a formal classification of CPM responders (i.e., i.e., inhibitors and facilitators) and non-responders. A formal classification was not performed because repeated measures of the TS without any intervention, necessary to determine the standard error of measurement and thus, to determine responders vs. non-responders, was not performed. These individual pain modulatory effects were qualitatively compared for PPT and HPT, i.e., no statistical tests on the proportions were performed.

To investigate the relationship between individual pain modulatory effects in a quantitative manner, Spearman’s rank correlations were performed between the parallel pain modulatory effects induced by the cold and the parallel pain modulatory effects induced by the sham water bath. The correlations were corrected for multiple comparisons using the false discovery rate.

# Results S1

# 1. Individual pain modulatory effects on PPT

Qualitative assessment of individual pain modulatory effects revealed positive, i.e., inhibitory, parallel pain modulatory effects on PPT during the cold water bath for 39 participants (79.6%) and during the sham water bath for 27 participants (55.1%, Figure S1a, Table S3). Of the 39 participants with positive pain modulatory effects during the cold water bath, 24 (61.5%) also showed positive pain modulatory effects during the sham water bath.

Negative pain modulatory effects, i.e., facilitatory, were detected for eight participants (16.3%) during the cold and for 19 participants (38.8 %) during the sham water bath. None of the eight participants with negative pain modulatory effect during the cold water bath also showed negative pain modulatory effects during the sham water bath.

Twenty-nine participants (59.2 %, Figure S1b, Table S3) showed positive and 16 participants (32.7 %) showed negative sham-controlled CPM effects on PPT. Thirteen (44.8 %) of the participants with positive sham-controlled CPM effect on PPT also showed positive sham-controlled CPM effect on HPT and 7 (43.8 %) of the participants with negative sham-controlled CPM effect on PPT also showed negative sham-controlled CPM effects on HPT.

# 2. Individual pain modulatory effects on HPT

Qualitative assessment of individual pain modulatory effects revealed positive, i.e., inhibitory, parallel pain modulatory effects on HPT during the cold water bath for 38 participants (77.6 %) and during the sham water bath for 41 participants (83.7%, Figure S1a, Table S3). Of the 38 participants with positive pain modulatory effects during the cold water bath, 33 (86.8 %) also showed positive pain modulatory effects during the sham water bath. Negative pain modulatory effects, i.e., facilitatory, were detected for seven participants (14.3%) during the cold and five participants (10.2%) during the sham water bath. Two (28.6%) of the seven participants with negative pain modulatory effect during the cold water bath also showed negative pain modulatory effects during the sham water bath. Twenty-three participants (46.9%, Figure S1b, Table S3) showed positive and 22 participants (44.9%) showed negative sham-controlled CPM effects on HPT.

Correlation analyses revealed that the parallel pain modulatory effects of the cold and the sham water bath on HPT were positively correlated, indicating that participants showing HPT increases during the painful CS also tend to show increases during the control CS (Figure S2). This was not the case for PPT.


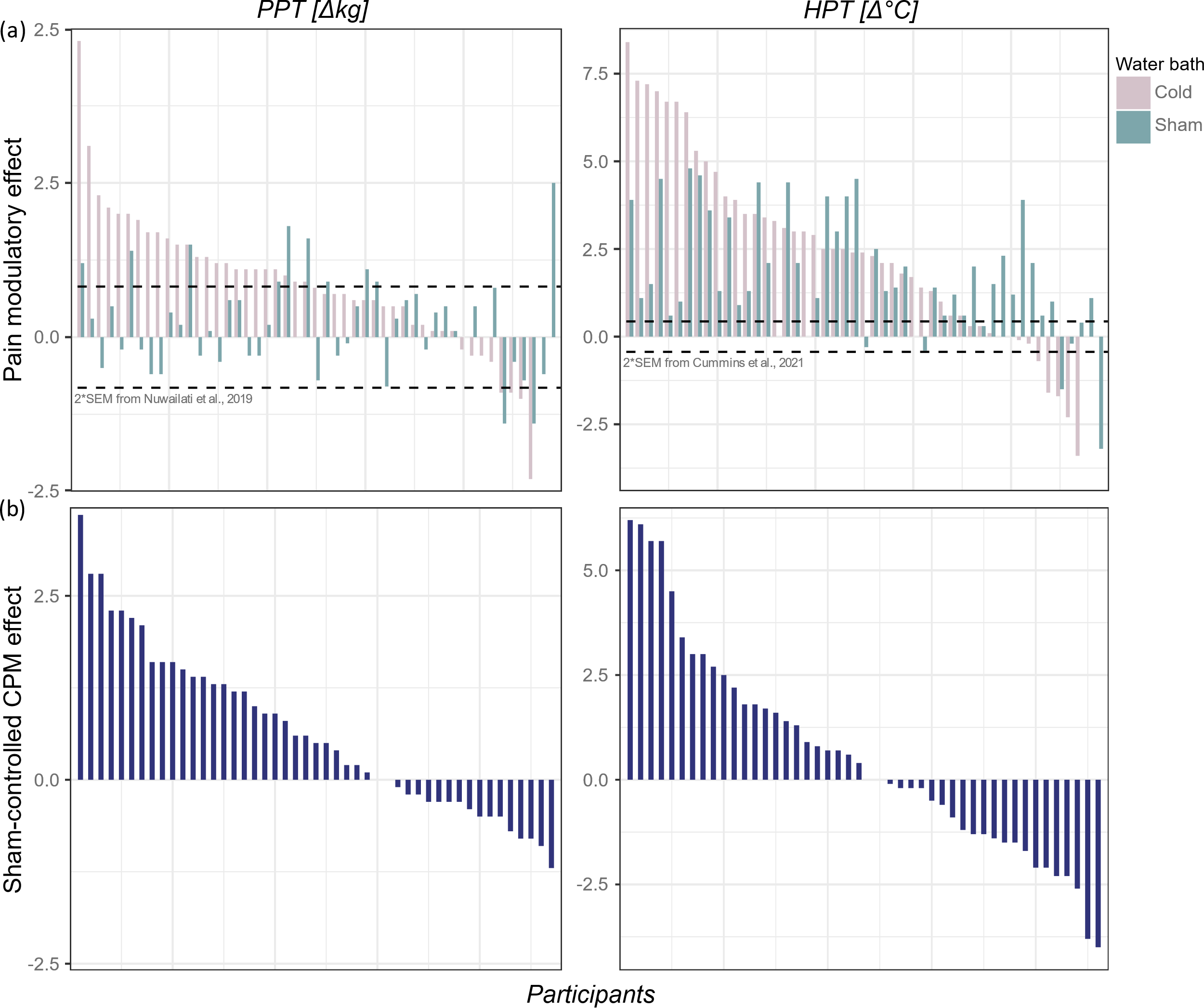


**Figure S1: Qualitative assessment of individual pain modulatory effects: a)** Parallel pain modulatory effect of the cold and sham water bath on PPT (left) and HPT (right) for each participant, absolute change of pain thresholds (bars) and ±2*SEM reported in Nuwailati et al., 2022 for PPT and Cummins et al., 2021 for HPT (dashed line). **b)** Parallel sham-controlled CPM effect on PPT (left) and HPT (right) for each participant, absolute change of pain threshold during cold minus absolute change of pain threshold during sham water bath (bars). CPM, conditioned pain modulation; HPT, heat pain threshold; PPT, pressure pain threshold; SEM, standard error of measurement.


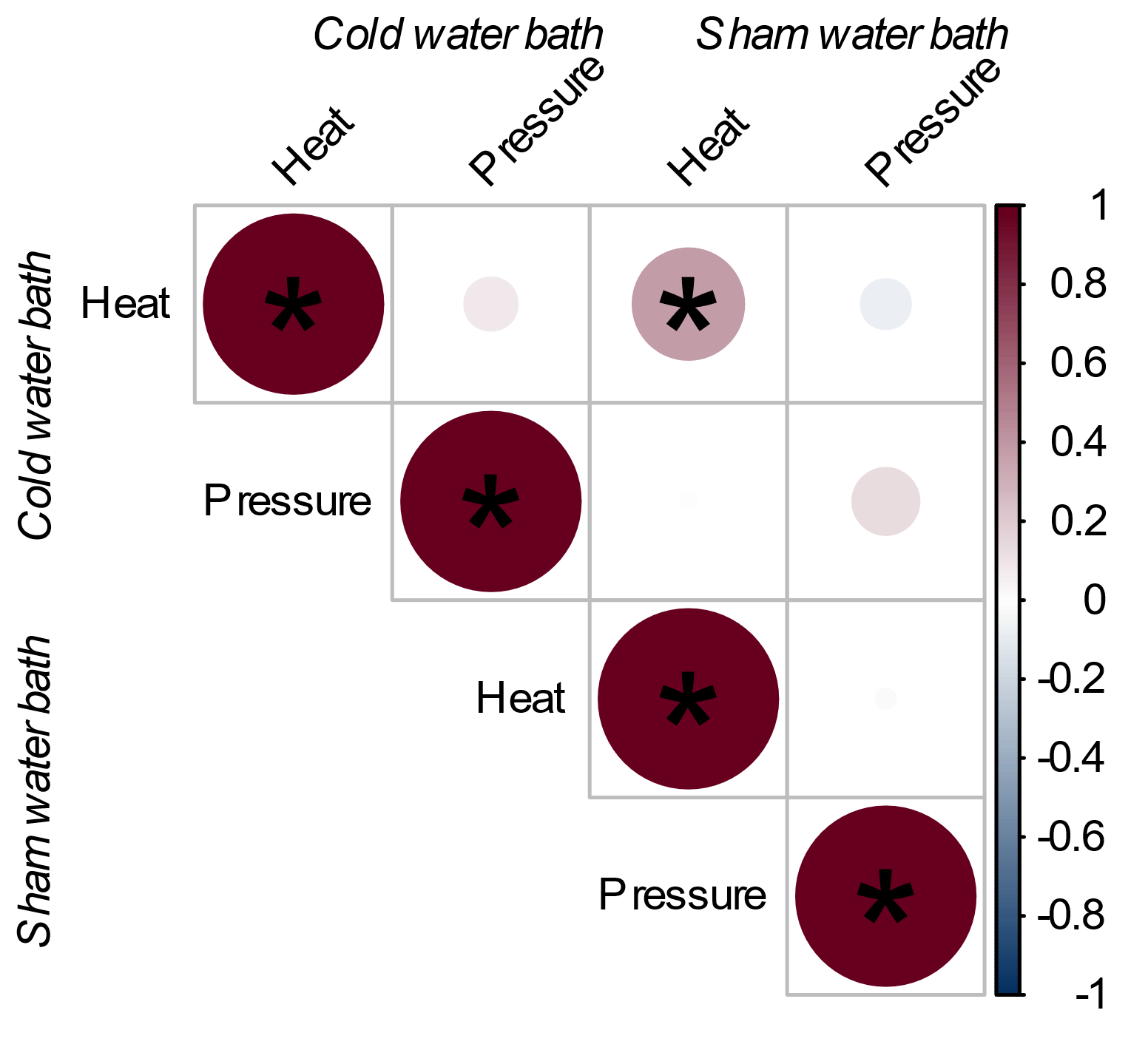


**Figure S2: Correlation analysis of parallel pain modulatory effects on pain thresholds**. * indicates p<0.05, FDR-corrected. Values are presented as Spearman's Rho. Size and color of the circles indicate correlation strength. Missing values: n=2 for heat and pressure during cold water bath, n=1 for heat during sham water bath

|  | Cold | | | Sham | | | Test statistics | | |
| --- | --- | --- | --- | --- | --- | --- | --- | --- | --- |
| Pain rating of water bath  median (interquartile range) [NRS] | 8.0 (2.00) | | | 0.0 (0.00) | | | V=1225, p<0.001 | | |
|  | Before | During | After | Before | During | After | Conditon | Timepoint | Interaction |
| HPT mean, SD [°C] | 43.2, SD=3.55^a^ | **45.7, SD=3.99**^a^ | **45.5, SD=3.65** | 43.4, SD=3.66 | **45.1, SD=3.50**^a^ | **45.1, SD=3.54**^a^ | F[238.1]=2.30 p=0.130 | *F[238.1]=57.98 p<0.001* |  |
| Pain modulatory effect  (before – during/after) |  | 2.5(5.9%),  SD=2.70 (6.63%) | 2.2 (5.2%),  SD=2.39 (5.81%) |  | 1.7 (4.2%),  SD=1.75 (4.14%) | 1.7 (4.1%),  SD=2.08 (4.94%) |  | | F[236.0]=1.47 p=0.232 |

**Table S1: HPT in the painful CS and control CS condition without exclusion of influential cases**. Italic font indicates significant main / interactions effects of the statistical models; bold font indicates significant post hoc tests, ^a^ indicates one missing value. Values are presented as mean, SD and, F and p values from linear mixed effects model. The cold water bath served as painful and the sham water bath as non-painful control CS. CS, conditioning stimulus; HPT, heat pain threshold; NRS, numerical rating scale; SD, standard deviation

**Table S2: Simulation-based power analysis of each model**. Bold font indicates targeted power of 1-β ≥0.8 was reached. HPT, heat pain threshold; PPT, pressure pain threshold; TSP, temporal summation of pain

|  | PPT Model | | | HPT Model | | | TSP Pressure Model | | | TSP Heat Model | | |
| --- | --- | --- | --- | --- | --- | --- | --- | --- | --- | --- | --- | --- |
| Number of simulated  Participants | 49 | 100 | 500 | 49 | 100 | 500 | 49 | 100 | 500 | 49 | 100 | 500 |
| Condition | 0.043 | 0.055 | 0.054 | 0.575 | **0.847** | **1** | 0.213 | 0.325 | **0.937** | 0.139 | 0.25 | **0.85** |
| Timepoint (During) | 0.28 | 0.502 | **0.999** | **1** | **1** | **1** |  |  |  |  |  |  |
| Timepoint (After) | 0.16 | 0.271 | **0.846** | **1** | **1** | **1** | 0.043 | 0.052 | 0.041 | 0.051 | 0.014 | 0.175 |
| Condition X Timepoint (During) | **0.815** | **0.979** | **1** | 0.069 | 0.118 | 0.469 |  |  |  |  |  |  |
| Condition X Timepoint (After) | 0.068 | 0.1 | 0.289 | 0.052 | 0.065 | 0.071 | 0.063 | 0.07 | 0.213 | 0.049 | 0.057 | 0.084 |

|  | PPT | | HPT | |
| --- | --- | --- | --- | --- |
|  | Positive | Negative | Positive | Negative |
| Cold | 39 | 8 | 38 | 7 |
| Sham | 27 | 19 | 41 | 5 |
| Sham-controlled | 29 | 16 | 23 | 22 |

**Table S3: Number of subjects with positive and negative parallel pain modulatory effects on pain thresholds.** Values are presented as absolute number of subjects with parallel positive and negative pain modulatory effects during the cold and the sham water bath and positive and negative parallel sham-controlled CPM effects. HPT, heat pain threshold; PPT, pressure pain threshold

|  | | **Segmentally applied** | | | | | **Extra-Segmentally applied** | | | | |
| --- | --- | --- | --- | --- | --- | --- | --- | --- | --- | --- | --- |
|  |  | **CS** | | **TS** | |  | **CS** | | **TS** | |  |
| **Reference** | **Cohort (n)** | **Type** | **Location** | **Type** | **Location** | **Pain modulatory effect** | **Type** | **Location** | **Type** | **Location** | **Pain modulatory effect** |
| Graven-Nielson et al. 1998 | 14 HC | 5% Hypertonic saline | Tibialis anterior muscle | PPT | Tibialis anterior (2 cm from infusion) | 1min post-infusion: 7.5%; 10min post-infusion: 8% | 5% Hypertonic saline | Tibialis anterior muscle | PPT | Brachioradialis muscle | 1min post-infusion: 25%, 10min post-infusion: 20% |
|  |  |  |  |  | Tibialis anterior (10 cm from infusion) | 1min post-infusion: -3%; 10min post-infusion: 4% |  |  |  |  |  |
|  |  |  |  |  | Ankle (frontal aspect) | 1min post-infusion: 11%, 10min post-infusion: 15% |  |  |  |  |  |
| Svensson et al. 1999 | 9 HC | 5% Hypertonic saline | Tibialis anterior muscle (left) | Electric pulses | Tibialis anterior muscle (Left) | -4% | 5% Hypertonic saline | Brachioradialis muscle (left) | Electric pulses | Tibialis anterior muscle (left) | 43% |
|  |  |  | Tibialis anterior muscle (right) |  |  | 26% |  | Brachioradialis muscle (right) |  |  | 37% |
| Defrin et al. 2010 | 7 HC | Contact heat | Forearm (ventral, 5cm away from TS) | Suprathreshold heat (rated on VAS 0-10) | Forearm (ventral, distal part) | -0.7, (-12.3%) | Contact heat | Lower leg (contralateral) | Suprathreshold heat (rated on VAS 0-10) | Forearm (ventral, distal part) | 1.6 (25.4%) |
|  |  |  | Upper Arm (ventral, 30cm away from TS) |  |  | 1.14 (18.6%) |  |  |  |  |  |
|  |  |  | Forearm (distal part, contralateral) |  |  | 1.26 (20.1%) |  |  |  |  |  |
| Oono et al. 2011 | 12 HC (men only) | Cold water | Hand | PPT | Flexor carpi radialis muscle | 16.7%,  SD=2.8 | Cold water | Hand | PPT | Tibialis anterior muscle | 66.3%,  SD=10.0 |
|  |  |  |  |  |  |  |  |  |  | Masseter muscle | 23.3%,  SD=4.3 |
|  |  |  |  | PPTol |  | 19.8%,  SD=2.4 |  |  | PPTol | Tibialis anterior muscle | 24.6%,  SD=2.3 |
|  |  |  |  |  |  |  |  |  |  | Masseter muscle | 32.6%,  SD=4.6 |
|  |  | Tourniquet | Upper arm | PPT |  | 15.1%,  SD=2.6 | Tourniquet | Upper arm | PPT | Tibialis anterior muscle | 43.4%,  SD=5.8 |
|  |  |  |  |  |  |  |  |  |  | Masseter muscle | 20.7%,  SD=3.4 |
|  |  |  |  | PPTol |  | 24.7%,  SD=4.9 |  |  | PPTol | Tibialis anterior muscle | 20.2%,  SD=2.7 |
|  |  |  |  |  |  |  |  |  |  | Masseter muscle | 20.5%,  SD=3.7 |
|  |  | Mechanical presssure | Craniofacial (head band) | PPT | Masseter muscle | 10.1%,  SD=2.7 | Mechanical presssure | Craniofacial (head band) | PPT | Tibialis anterior muscle | 29.1%,  SD=5.7 |
|  |  |  |  |  |  |  |  |  |  | Flexor carpi radialis muscle | 13.8%,  SD=4.7 |
|  |  |  |  | PPTol |  | 24.4%,  SD=4.9 |  |  | PPTol | Tibialis anterior muscle | 18.7%,  SD=4.8 |
|  |  |  |  |  |  |  |  |  |  | Flexor carpi radialis muscle | 15.0%,  SD=3.4 |

**Table S4: Literature overview of pain modulatory effects of segmentally applied and extra-segmentally applied CS**. The pain modulatory effect is given in absolute values and/or %-change and standard deviation (if reported), positive values indicate hypoalgesia. CPM, conditioned pain modulation; CS, conditioning stimulus; HC, healthy controls; PPT, pressure pain threshold; PPTol, pressure pain tolerance threshold; SD, standard deviation; TS, test stimulus.
